# Hernia

**Published:** 1872-07

**Authors:** D. L. D. Sheldon


### HERNIA.

D. L. D. SHELDON, M. D.

BURST, BREACH, RUPTURE AND HERNIA; each of which names have been used to represent a disability existing in both sexes, at all ages and in all the various conditions of life, from which a larger proportion of humanity are now suffering, than from all other diseases combined under actual treatment; notwithstanding its frequency, and the extreme danger by strangulation, it is less understood by physicians than almost any other disease. Such ignorance is not a shame merely, but rank close with crime; for thousands of lives are thus continually being sacrificed. A majority of physicians attempt to shield their incapacity in this branch of the profession, by transferring their patients to *apothecaries*, *druggists* and *truss-dealers*, who know little or nothing of the anatomy of hernia, or the manner by which the protrusile parts make their escape from the abdominal cavity; thus, their ignorance, to certain extent, is kept within themselves, at the expense of destruction to their patient. A physician would hardly be considered sane, were he to call in a druggist or truss dealer to consult in a case of fever or fracture, merely because he sold such articles as are used in their treatment; yet it would be no less absurd to transfer a case of hernia to them.

This disease, technically named hernia, is constituted by a protrusion from the abdominal cavity; the parts protruding are generally *intestine*, often accompanied by *omentum*, (commonly termed caul); nearly all the other organs within the abdominal cavity of both sexes are very unfrequently capable of protrusion. The tumor generally exists in the region of the groin but many times at the naval, and much less frequent at other points. When at the groin, it is called inguinal hernia; this variety is far more prevalent than all the others combined. The protrusile parts in this variety, seldom pass, as is generally supposed, *directly* through the parieties of the abdomen, but in an *indirect* or *oblique* manner.

The space through which they pass is called the inguinal canal: its diameter in an adult is less than half an inch, its length less than two inches, and its angle about forty degrees with the plane of the pubis, (or cross bone), where it terminates; nature created this canal expressly for another purpose, but apparently, contrary to her design, from certain causes, the protruding parts escape through it. I presume it will now be readily perceived, that the large and inappropriate truss-pads, commonly used by truss dealers, and by the unprogressive of the profession, are not what is required to close the mouth of this narrow canal. The object of truss pressure in this variety, is to bring and retain the anterior and posterior walls of this canal in contact, particularly at its commencement within the abdomen, thereby checking the protrusion into the canal. If this pressure be properly applied, and retained a proper length of time, a cure in very many cases will be perfected by the permanent closure of this canal. It will now be understood how I have checked protrusion when the tumor had previously extended *ten to twelve* inches in length, and *twenty* in circumference, with a hard compress, less than one and a quarter inches in diameter and less than two inches in length.

Truss inventors being mostly ignorant of the anatomy of hernia, or the manner by which protrusion is effected, have arranged their trusses more particularly for closing the terminal aperture of the canal, by pressing upon the pubis, a point where pressure is to a great extent destructive to generative power; seeming to disregard the important point of closing the mouth of the canal, but leave it to the mercy of the protrusile parts; the few cures from such treatment is generally accidental.

As a rule, no person understands this branch, unless a graduate from some properly recognized medical institute. Were the medical profession equal to their duty, those afflicted with this disease would realize the necessity and importance of a proper truss, well adjusted, to secure them from speedy death by strangulation; the destructive agent which is generally unrecognized until the destroyer has secured its victim.

